# Molecular Imaging of Lower Extremity Peripheral Arterial Disease: An Emerging Field in Nuclear Medicine

**DOI:** 10.3389/fmed.2021.793975

**Published:** 2022-01-12

**Authors:** Mitchel R. Stacy

**Affiliations:** ^1^Center for Regenerative Medicine, The Research Institute at Nationwide Children's Hospital, Columbus, OH, United States; ^2^Division of Vascular Diseases and Surgery, Department of Surgery, The Ohio State University College of Medicine, Columbus, OH, United States

**Keywords:** peripheral arterial disease (PAD), positron emission tomography (PET), single photon emission computed tomography (SPECT), computed tomography, molecular imaging

## Abstract

Peripheral arterial disease (PAD) is an atherosclerotic disorder of non-coronary arteries that is associated with vascular stenosis and/or occlusion. PAD affecting the lower extremities is characterized by a variety of health-related consequences, including lifestyle-limiting intermittent claudication, ulceration of the limbs and/or feet, increased risk for lower extremity amputation, and increased mortality. The diagnosis of lower extremity PAD is typically established by using non-invasive tests such as the ankle-brachial index, toe-brachial index, duplex ultrasound, and/or angiography imaging studies. While these common diagnostic tools provide hemodynamic and anatomical vascular assessments, the potential for non-invasive physiological assessment of the lower extremities has more recently emerged through the use of magnetic resonance- and nuclear medicine-based approaches, which can provide insight into the functional consequences of PAD-related limb ischemia. This perspectives article specifically highlights and discusses the emerging applications of clinical nuclear medicine techniques for molecular imaging investigations in the setting of lower extremity PAD.

## Introduction

Peripheral arterial disease (PAD) is an atherosclerotic disease affecting non-coronary arteries that is associated with vascular stenosis and/or occlusion. Lower extremity PAD is defined as an atherosclerotic obstruction affecting arterial inflow at any vascular site from the aortoiliac segments to the pedal arteries. This atherosclerotic condition negatively impacts lower extremity functional capacity and quality of life by reducing blood flow, perfusion, and oxygen delivery to skeletal muscle of the calves and feet, as well as through resulting in reductions in calf muscle area and increases in fatty infiltration of muscle and muscle fibrosis (1). Furthermore, PAD of the lower extremities is associated with increased morbidity and mortality, with PAD now representing the third leading cause of atherosclerotic morbidity, ranking only behind coronary artery disease and stroke (2). More than 12 million Americans (3) and >230 million people worldwide (2) are estimated to have PAD; however, PAD continues to be largely underdiagnosed and undertreated (4) due to a lack of screening in the general population (5). Therefore, accurate, non-invasive tools are critical for screening, diagnosing, and monitoring PAD patients to improve risk stratification, facilitate treatment options, and better quantify responses to treatment.

Standard non-invasive clinical tools such as the ankle-brachial index (ABI), toe-brachial index (TBI) are common for screening patients with suspected PAD and for evaluating patients during clinic visits; however, ABI and TBI are arterial hemodynamic measures that can be misleading in the setting of medial calcification and incompressible vessels, which often occurs in the setting of diabetes mellitus (DM). Duplex ultrasound can also be utilized to detect the presence of arterial obstruction, but it also is limited in its application by only offering assessment of arterial patency and flow. Computed tomography (CT) and magnetic resonance (MR) angiographic approaches exist for assessing patients with PAD, however, these methods also only evaluate arterial patency. Catheter-based angiography continues to be the gold standard for diagnosing PAD, but requires arterial catheterization, only evaluates arterial patency, and is only recommended for patients who are undergoing endovascular revascularization procedures (6).

Compared with other more established clinical PAD imaging tools that primarily measure vascular anatomy and hemodynamics, the nuclear imaging modalities of single photon emission computed tomography (SPECT) and positron emission tomography (PET) may offer unique insight into PAD pathophysiology and play an important role in the non-invasive evaluation of both lower extremity arteries and skeletal muscle in the setting of PAD. Historically, nuclear imaging of lower extremity skeletal muscle perfusion first emerged in the 1940s (7, 8), with very little incremental change or application of nuclear imaging in the setting of PAD until several decades later following the clinical integration of scintigraphy imaging (9, 10). However, the last decade has seen an emergence of new applications and methods for using nuclear imaging modalities to quantify lower extremity pathophysiology in PAD. While novel MR- (11–15) and ultrasound-based (16, 17) imaging approaches have also emerged in recent years that offer physiological evaluation of lower extremity skeletal muscle, these techniques are beyond the scope of this article. This perspectives article will discuss current advances and emerging applications of clinical nuclear imaging modalities in recent years in the setting of lower extremity PAD, with particular focus on perfusion imaging and molecular imaging of atherosclerosis and vascular inflammation.

## Imaging of Lower Extremity Skeletal Muscle Perfusion

Impairment of lower extremity skeletal muscle perfusion is a hallmark of PAD and a primary contributor to functional decrements that are common for many patients with PAD. Severe perfusion abnormalities can lead to the onset of critical limb ischemia (CLI), the severe manifestation of PAD, which is characterized by a high risk for non-healing wounds, lower extremity amputation, and death (3). While perfusion deficits are a known contributor to symptom severity and morbidity and mortality in PAD, an ongoing clinical challenge is the lack of a validated vascular test for detecting and monitoring these common perfusion abnormalities. Indeed, this challenge was recently highlighted in a scientific statement by the American Heart Association (3). The establishment of an accurate non-invasive approach for quantifying regional lower extremity muscle perfusion in both the calves and the feet could greatly facilitate diagnosis of PAD and CLI, clinical decision making, and monitoring of responses to treatments directed at wound healing and limb salvage.

**Figure 1:**
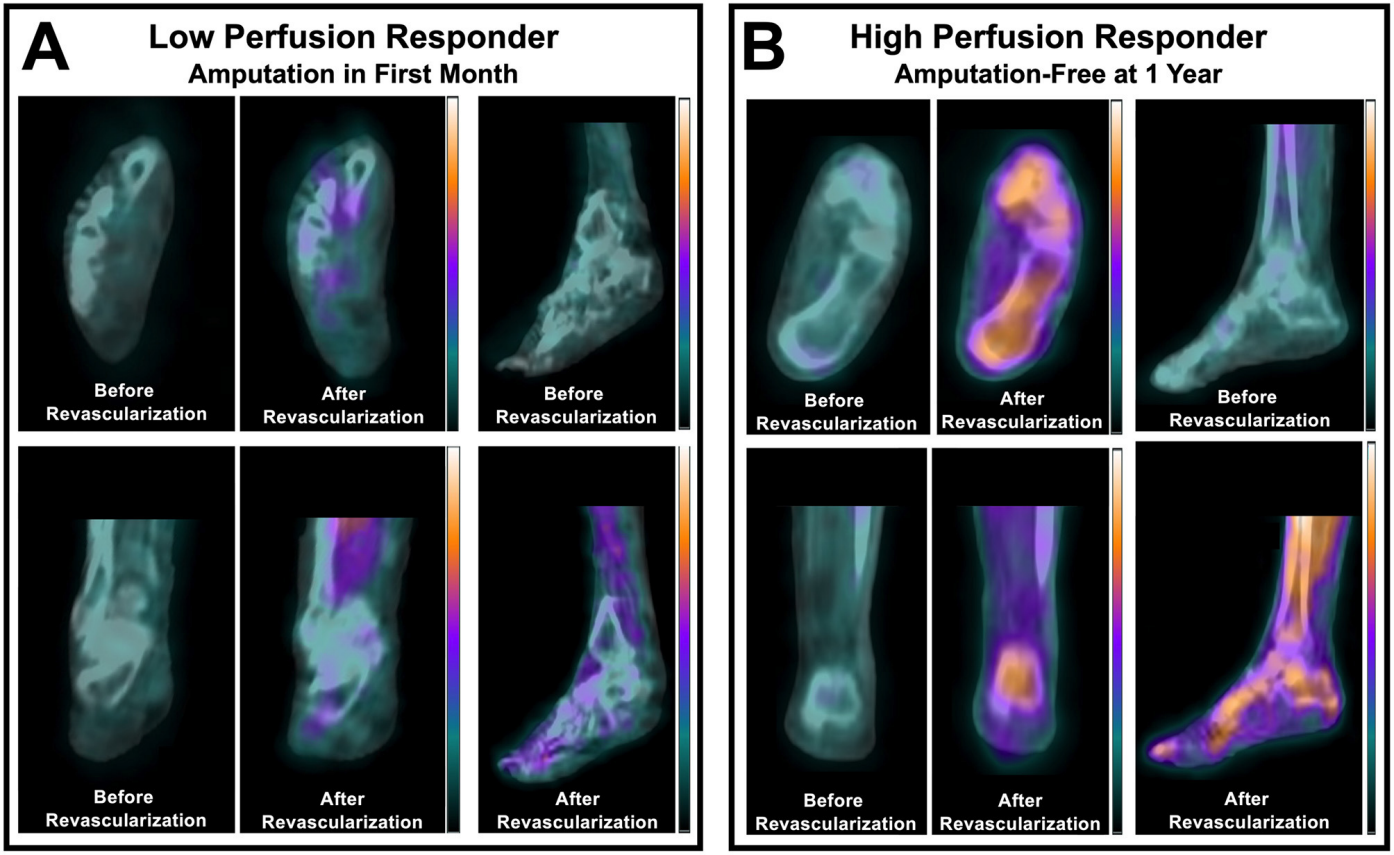
SPECT/CT imaging of the perfusion response to lower extremity revascularization in patients with CLTI. Non-invasive imaging identified differential perfusion responses to peripheral balloon angioplasty in **(A)** a patient with a low perfusion response who underwent amputation within one month, and **(B)** a patient who had a high relative perfusion response and remained amputation-free for one year after intervention. Adapted from Chou et al. (22). For a complete description of perfusion imaging methodology, please refer to Chou et al. (22).

Recently published nuclear imaging studies in the last 3 years focused on evaluating lower extremity perfusion in PAD patients have demonstrated the utility of hybrid SPECT/CT imaging for non-invasively detecting abnormalities in microvascular perfusion within the feet of diabetic patients with CLI. Specifically, SPECT/CT imaging with technetium-99m (^99m^Tc)-tetrofosmin, a standard myocardial perfusion radionuclide that is retained based on mitochondrial membrane potential and tissue viability (18), has been shown to allow for evaluation of relative perfusion defects within specific vascular territories, or angiosomes, of the feet (19). Additionally, this approach has revealed utility for detecting resting differences in regional foot perfusion between CLI patients and healthy control subjects (19), assessing tissue viability that corresponds with the subsequent level of amputation (20), and quantifying regional improvements in relative perfusion within the foot that occurs in response to endovascular revascularization (21, 22). Most recently and importantly, perfusion imaging of the feet using SPECT/CT imaging has also demonstrated prognostic value for predicting risk for lower extremity amputation in patients with CLI who underwent endovascular revascularization for limb salvage, where patients who were high perfusion responders to revascularization experienced greater limb salvage success compared to those who were categorized as low perfusion responders (Figure 1) (22). Along with three-dimensional SPECT perfusion imaging showing promising results for evaluating resting perfusion and the response to revascularization in patients with CLI, two-dimensional scintigraphy imaging studies have also been used in recent years to evaluate the response to angiogenesis-promoting cell therapies in the setting of CLI, where resting uptake of ^99m^Tc-tetrofosmin significantly increased 4 weeks after transplantation of bone marrow mononuclear cells in the calves and feet (23).

Beyond the application of nuclear imaging of perfusion in the setting of CLI, other research teams have also demonstrated that SPECT-derived measures of resting calf muscle perfusion may possess prognostic value for predicting risk for major cardiovascular adverse events in the PAD patient population (24). Additionally, ^99m^Tc-tetrofosmin SPECT/CT perfusion imaging of the calves has recently been shown to significantly correlate with both exercise capacity and cardiovascular fitness in non-PAD clinical populations, thus suggesting that nuclear imaging of calf muscle perfusion could serve as a non-invasive correlate for investigating lower extremity physiological adaptations to exercise training programs in the setting of PAD (25). The latter study may be of particular relevance for the PAD community considering the 2017 approval for national coverage of supervised exercise therapy for symptomatic PAD by the Centers for Medicare and Medicaid Services (26). Furthermore, this study highlighted the feasibility and practicality of additional perfusion imaging of the lower extremities in patients already undergoing clinically-indicated myocardial perfusion imaging, where quantitative assessment of calf muscle perfusion reserve was achieved without the need for additional radionuclide injections, additional stress testing, or additional time in clinic (25). Thus, patients could undergo simultaneous screening of lower extremity perfusion at the time of cardiac imaging, which could potentially allow for early identification of perfusion abnormalities in asymptomatic/undiagnosed PAD patients.

While several noteworthy developments have been made in the past decade using SPECT- and scintigraphy-based perfusion imaging methods for the PAD patient population, interestingly, similar advancements have not been made with regard to PET perfusion imaging of lower extremity muscle perfusion in PAD aside from a small number of studies published in past decades that used oxygen-15 (^15^O)-water to assess calf muscle perfusion (27–29). ^15^O-water, which is a freely diffusible and metabolically inert radionuclide that represents the gold standard for quantitative perfusion (30), has specifically demonstrated its potential for providing absolute measures of lower extremity muscle perfusion (i.e., ml/min/g) (27), which has not been previously shown using other PET radioisotopes or SPECT imaging techniques. Thus, the opportunities for growth and application of PET-based perfusion methodologies is apparent and could ultimately enable enhanced quantitative evaluation of skeletal muscle perfusion in the setting of PAD.

## Molecular Imaging of Peripheral Artery Inflammation and Atherosclerosis

PET imaging of arterial inflammation and atherosclerosis has also garnered attention in the cardiology community in the past decade, with a large majority of published studies focusing on the application of fluorine-18 (^18^F)-fluorodeoxyglucose (FDG) and ^18^F-sodium fluoride (NaF) for molecular imaging of inflammation and atherosclerotic disease progression in the carotid arteries, coronary arteries, and aorta (31). ^18^F-FDG in particular continues to be one of the most widely used PET-based approaches for assessing arterial inflammation due to it being a glucose analog that possesses an affinity for metabolically active macrophages that are present in inflamed atherosclerotic plaques (32–34). Despite the overwhelming existing body of literature focused on PET imaging of various arterial networks, to date, PET imaging of inflammation and atherosclerosis remains relatively understudied in the setting of lower extremity PAD. In non-PAD patient populations, ^18^F-FDG PET/CT imaging has been found to be useful for evaluating vascular inflammation in the lower extremities, and these PET-derived measures significantly correlate with measures of peripheral vascular stiffness (35). Additionally, serial ^18^F-FDG PET/CT has shown utility for non-invasively detecting statin-induced reductions in inflammation within the femoral arteries of patients with dyslipidemia (36). However, only a limited number of recent studies have specifically evaluated the utility of ^18^F-FDG PET as a tool for quantifying arterial inflammation in patients with PAD. One recent study by Jiang et al. (37) applied serial ^18^F-FDG PET/CT imaging in PAD patients before and after sonodynamic therapy to the femoropopliteal artery, which demonstrated that PET/CT imaging could detect and quantify therapeutic reductions in arterial inflammation in the setting of PAD. Another recent study focused on evaluating the utility of a combined ^18^F-FDG PET and MR imaging approach for simultaneous assessment of both plaque morphology and arterial inflammation in a small sample size of PAD patients, but did not find any significant correlations between ^18^F-FDG arterial uptake and histological measures of arterial inflammation (38). Thus, ongoing work focused on targeted imaging of arterial inflammation in the setting of PAD is warranted to fully elucidate the potential role of PET imaging as a non-invasive biomarker of PAD-induced inflammation.

**Figure 2:**
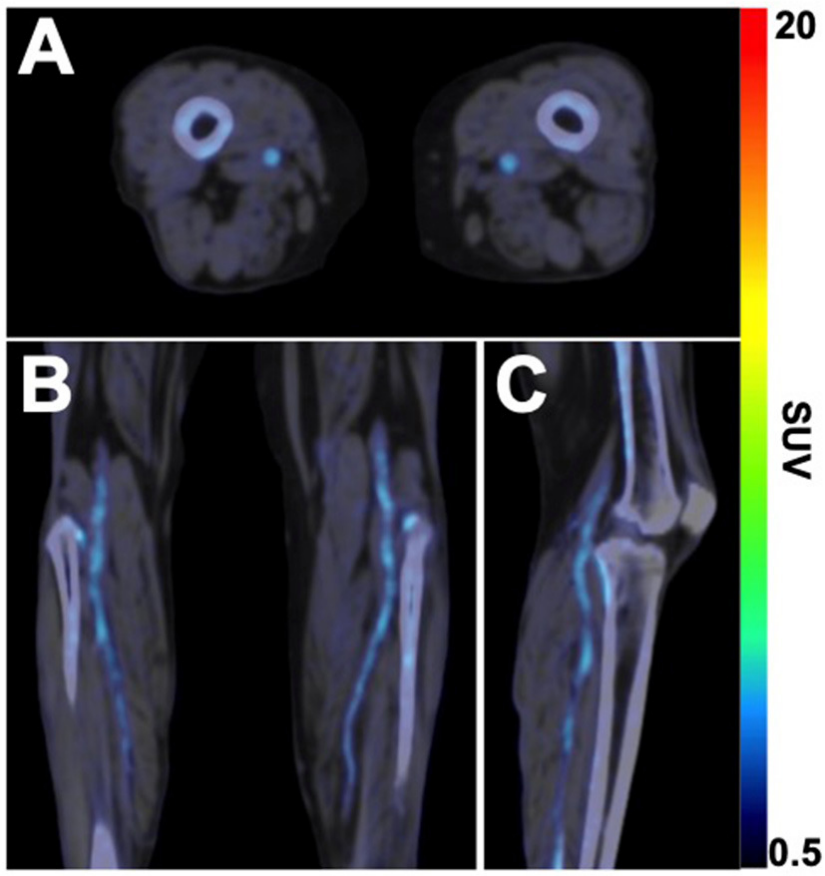
^18^F-NaF PET/CT imaging of active microcalcification in PAD. **(A)** Axial, **(B)** coronal, and **(C)** sagittal fused ^18^F-NaF PET/CT images acquired in a 63-year old female patient with CLTI and type 2 diabetes mellitus demonstrates the active process of microcalcification in above- and below-the-knee arteries. Non-contrast CT images were acquired for attenuation correction of PET data, followed by static PET imaging of the lower extremities 75 min after intravenous administration of ^18^F-NaF (296 MBq). SUV, standardized uptake value.

In addition to ^18^F-FDG, ^18^F-NaF has more recently emerged as a radionuclide of interest for studying the active process of atherosclerosis. Historically, ^18^F-NaF was originally approved in the 1960s and used for targeted imaging of bone remodeling due to its high affinity for hydroxyapatite, the mineral form of calcium apatite (39). However, in the last 10–15 years, a large body of cardiovascular literature has emerged that has explored the utility of ^18^F-NaF as a tool for quantifying the active process of vascular microcalcification (31). As with ^18^F-FDG, ^18^F-NaF has only recently been applied and investigated in the setting of lower extremity PAD. Initial studies using ^18^F-NaF in PAD patients approximately 10 years ago demonstrated the feasibility of using this radionuclide for PET imaging of the lower extremities (40) and revealed that femoral artery uptake of ^18^F-NaF was strongly associated with cardiovascular risk factors and high-risk profiles for cardiovascular events (41). Following an ~7 year period of time passing without a single study published in this field, an increasing number of studies have emerged in the last 2 years using ^18^F-NaF to evaluate peripheral atherosclerosis in patients with PAD. These studies have demonstrated that arterial uptake of ^18^F-NaF is significantly higher in non-lower extremity arteries of PAD patients compared to non-PAD patients (42) and that femoral artery ^18^F-NaF uptake is significantly associated with various modifiable cardiovascular risk factors (i.e., cholesterol, triglycerides, HbA1c) (43), thus suggesting that ^18^F-NaF PET/CT imaging could be used in the future for non-invasively monitoring the response to treatments focused on cholesterol reduction and/or glucose management. Pictured in Figure 2 is a representative example of ^18^F-NaF PET/CT imaging in a patient with DM and CLI, which demonstrates the ability of ^18^F-NaF to detect active disease progression in the lower extremities.

Other additional applications of ^18^F-NaF in the setting of PAD over the past 2 years have demonstrated the feasibility of using ^18^F-NaF PET/CT imaging to evaluate active microcalcification of occlusive lower extremity aneurysms (44), quantify the inflammatory response to lower extremity balloon angioplasty and predict risk for vascular restenosis after peripheral interventions (45), and evaluate the role of arterial inflammation in promoting systemic vascular calcification (46). Thus, the applications for ^18^F-NaF PET/CT are rapidly evolving, with numerous future research directions on the horizon for molecular imaging of atherosclerosis in patients with PAD.

## Discussion

Nuclear and molecular imaging of PAD is an emerging field that provides numerous opportunities for physiological investigation into this traditionally underdiagnosed and undertreated disease. Recent studies have demonstrated that SPECT/CT perfusion imaging may enable the screening, diagnosis, and monitoring of responses to treatment (21, 22, 25), while PET/CT imaging may provide novel opportunities for molecular imaging of atherosclerosis and vascular inflammation (47), which to date, have remained relatively understudied in the setting of lower extremity PAD. Additionally, these hybrid nuclear imaging approaches that utilize CT can offer simultaneous evaluation of calcium burden in the lower extremities that is not possible with conventional vascular imaging techniques (20). While nuclear imaging approaches in the setting of PAD remain exploratory in nature, these imaging techniques could potentially assist with screening for and diagnosis of regional perfusion abnormalities related to PAD and severity of PAD, which could subsequently assist clinicians by guiding targeted revascularization procedures or evaluating the response to revascularization. Additionally, PET/CT imaging of arterial inflammation and active atherosclerosis may assist clinicians by detecting regions of active disease, thereby guiding endovascular therapy or monitoring of problematic lesions. Currently, hemodynamic tools such as ABI, TBI, and Doppler ultrasound are a mainstay of screening for PAD due to their relative efficiency and cost-effectiveness; however, perfusion imaging with nuclear techniques has proven to provide further physiological information beyond traditional hemodynamic assessment by detecting the specific anatomical region of underlying tissue ischemia, thus potentially setting the stage for their use during PAD diagnosis and treatment planning. Collectively, nuclear imaging techniques advance the non-invasive evaluation of PAD beyond traditional means by offering quantitative regional analysis of vascular and muscle physiology, whereas traditional non-invasive vascular diagnostic tools have primarily focused on hemodynamic (e.g., ABI, TBI, ultrasound) or structural (e.g., angiography) assessments of the lower extremities. It's important to note that while SPECT/CT and PET/CT imaging have demonstrated potential in PAD, the recent emergence of PET/MR imaging may also provide additional opportunities for partnering high-sensitivity molecular (PET) and high-resolution structural (MR) imaging in the setting of lower extremity PAD (38, 48).

Given the multifactorial nature of PAD-related complications, ongoing advancements in nuclear medicine and molecular imaging should facilitate development of novel imaging strategies that are capable of targeting the underlying pathophysiology associated with lower extremity PAD and enable serial monitoring of physiological responses to medical treatment. Specifically, advancements with cadmium zinc telluride (CZT) SPECT systems and whole-body PET cameras may offer new approaches for quantifying absolute perfusion of lower extremity skeletal muscle beyond what has been previously accomplished with conventional ^15^O-water PET imaging. Expanded application of ^18^F-FDG and ^18^F-NaF to the lower extremities, along with other developing radionuclides meant for atherosclerosis and thrombosis targeted imaging, could also allow for novel opportunities to investigate mechanisms associated with PAD disease progression and non-invasively detect occlusive peripheral thrombi (49–53). Additionally, the use of multi-tracer imaging of different physiological processes in the lower extremities could theoretically be achieved with SPECT imaging of radionuclides that possess distinctly different gamma ray energy photopeaks, or with PET imaging by staggering injection times of short half-life radionuclides; however, the advantages and disadvantages associated with increased radiation exposure for patients receiving multiple radionuclide injections in a single imaging session would need to be carefully considered. Beyond the various clinical investigations focused on nuclear imaging of PAD, a large number of pre-clinical studies have been published in recent years that also highlight ongoing developments in the field of molecular imaging that could possess translational potential for PAD patients. These studies have utilized large and small animal models of atherosclerosis and hindlimb ischemia to validate novel SPECT- and PET-based approaches directed at perfusion (54) and angiogenesis targeted imaging (55–57), which continue to be the primary areas of pre-clinical PAD research. Overall, molecular imaging of lower extremity PAD remains a developing and exciting field of research that should provide novel insight into PAD pathophysiology and eventually expand the repertoire of non-invasive tests available to vascular medicine specialists.

## Data Availability Statement

The original contributions presented in the study are included in the article/supplementary material, further inquiries can be directed to the corresponding author/s.

## Ethics Statement

The studies involving human participants were reviewed and approved by Nationwide Children's Hospital Institutional Review Board. The patients/participants provided their written informed consent to participate in this study.

## Author Contributions

The author confirms being the sole contributor of this work and has approved it for publication.

## Funding

This work was supported by National Institutes of Health award R01 HL135103.

## Conflict of Interest

The author declares that the research was conducted in the absence of any commercial or financial relationships that could be construed as a potential conflict of interest.

## Publisher's Note

All claims expressed in this article are solely those of the authors and do not necessarily represent those of their affiliated organizations, or those of the publisher, the editors and the reviewers. Any product that may be evaluated in this article, or claim that may be made by its manufacturer, is not guaranteed or endorsed by the publisher.
